# Individual differences in adult handwritten spelling-to-dictation

**DOI:** 10.3389/fpsyg.2013.00402

**Published:** 2013-07-16

**Authors:** Patrick Bonin, Alain Méot, Séverine Millotte, Christopher Barry

**Affiliations:** ^1^Institut Universitaire de France; ^2^LEAD-CNRS, University of BourgogneDijon, France; ^3^LAPSCO-CNRS, University Blaise PascalClermont-Ferrand, France; ^4^University of EssexEssex, UK

**Keywords:** spelling, writing, dictation, individual differences, dual-route model, word frequency, phoneme-to-grapheme consistency

## Abstract

We report an investigation of individual differences in handwriting latencies and number of errors in a spelling-to-dictation task. Eighty adult participants wrote a list of 164 spoken words (presented in two sessions). The participants were also evaluated on a vocabulary test (Deltour, 1993). Various multiple regression analyses were performed (on both writing latency and errors). The analysis of the item means showed that the reliable predictors of spelling latencies were acoustic duration, cumulative word frequency, phonology-to-orthographic (PO) consistency, the number of letters in the word and the interaction between cumulative word frequency, PO consistency and imageability. (Error rates were also predicted by frequency, consistency, length and the interaction between cumulative word frequency, PO consistency and imageability.) The analysis of the participant means (and trials) showed that (1) there was both within- and between-session reliability across the sets of items, (2) there was no trade-off between the utilization of lexical and non-lexical information, and (3) participants with high vocabulary knowledge were more accurate (and somewhat faster), and had a differential sensitivity to certain stimulus characteristics, than those with low vocabulary knowledge. We discuss the implications of these findings for theories of orthographic word production.

How is the spelling of the words that we know derived to produce a written trace on a sheet of paper? Any theory of written spelling must account for how the cognitive system implemented in the brain goes from an auditory input, a pictured object, or an idea to the muscular realization of the spelling response. In the present study, we focused on handwritten spelling-to-dictation and addressed the general issue of individual differences in this verbal skill. This issue has recently been addressed in visual word recognition (Yap et al., 2012) but it has never been addressed in word spelling production. In order to illustrate the different issues that we wish to investigate here, we will first sketch a dual-route view of the spelling process in adults based on the recent proposals of Purcell et al. (2011) and Rapp et al. (2002).

The dual-route view is the dominant view of word spelling. It posits that there are two routes available for spelling familiar words: a lexical and a non-lexical route. The lexical route permits the spelling of known words through the retrieval of lexical knowledge from the output orthographic lexicon whereas the non-lexical route makes use of sublexical knowledge to provide the spelling of unknown words and non-words. This view is supported by various lines of evidence (Tainturier and Rapp, 2001 for a review).

Within the dual-route architecture, there are central and peripheral components. The central components consist of orthographic long-term memory, phoneme–grapheme conversion, and orthographic working memory. The word spellings that people know are stored in orthographic long-term memory. It is generally assumed that orthographic wordform representations are retrieved from the semantic codes that are activated from the auditory processing of the heard word. Word frequency is assumed to affect orthographic wordform retrieval within the lexical route and its influence on spelling performance is taken as an index of the mobilization of this route. Word frequency effects correspond to the observation that high-frequency words are produced faster and more accurately than low-frequency words (e.g., Delattre et al., 2006). The spelling of words can also be assembled from the phonological codes derived from auditory processing by the involvement of a non-lexical conversion procedure. Traditionally, it has been proposed that phoneme-grapheme units are involved in the conversion process (Tainturier and Rapp, 2001).

The ambiguity of the relationships between sound and spelling units is generally operationalized with the PO consistency variable^1^. This variable affects spelling performance with the result that inconsistent words, and low-frequency words in particular, take longer to produce than consistent words (Bonin and Méot, 2002), and it is therefore taken as an index of the involvement of the non-lexical route. Some accounts have explained consistency effects in terms of a conflict between the different individual graphemes that, in the case of irregular words, are activated by the non-lexical and lexical routes at the grapheme level, unlike in the case of regular words (e.g., Rapp et al., 2002). Abstract individual letter representations are activated at the level of orthographic-working memory (Rapp and Dufor, 2011) which maintains letter identity and order information active for processing by peripheral components. Word length effects in spelling are assumed to result from the involvement of this working memory system. The peripheral processes are responsible for the generation of a written trace in handwritten spelling. It is assumed that abstract letter representations form the basis for the processing stages of allographic conversion (the choice of case and specific writing style), letter shape assignment, and motor muscular programming and execution of the effector-specific muscle movements required to output letters. There is evidence supporting the idea of interactions between different central components (e.g., Roux et al., 2013) and between central and peripheral processes (e.g., Delattre et al., 2006).

There are only a few on-line studies of the word spelling performance of healthy adults (Bonin and Méot, 2002; Bonin et al., 2004). In the Bonin et al. study (2004), a multiple regression approach was used to investigate the determinants of written spelling latencies corresponding to individual words. The participants had to write down, on a graphic tablet, bare nouns that were presented orally. The reliable predictors of spelling latencies were acoustic duration, objective cumulative word frequency, PO consistency and word length. Bonin and Méot (2002) also found a reliable interaction between word frequency and PO consistency in spelling-to-dictation latencies: the consistency effect was larger for low-frequency words than for high-frequency words. As claimed above, this finding accords with the prediction of the dual-route view because it is assumed that consistency effects are the result of a competition between the outcomes of the lexical and non-lexical routes, respectively (Tainturier and Rapp, 2001). Finally, and also in line with the dual-route view of spelling, Bonin and Méot (2002) found that imageability (a variable assumed to index semantic code activation, Evans et al., 2012) interacted reliably with word frequency and PO consistency, with the result that the joint influence of word frequency and PO consistency was most pronounced on words of low imageability. Overall, the findings were consistent with the dual-route view which posits that spelling to dictation requires the interactive involvement of different types of knowledge: lexical, sublexical, and semantic knowledge.

In word reading, where the dual-view has proved to be very influential (Coltheart et al., 2001), it is generally assumed that the two routes differ in their processing characteristics. It has sometimes been assumed that the non-lexical route is slower and less automatized than the lexical route (e.g., Paap and Noel, 1991). Importantly to note for the purposes of our study is the claim that the non-lexical route might be under *strategic control*, with the result that its involvement in the processing of words might be emphasized or de-emphasized under specific conditions. Certain word reading studies have tried to identify reading profiles according to the dominant reliance on the lexical or non-lexical route. According to these studies, one type of reader relies more on the lexical route than on the non-lexical route whereas another type relies more heavily on the non-lexical route (e.g., Baron and Strawson, 1976; Weekes, 1994). However, there is as yet no clear evidence in support of this view (Burt and Heffernan, 2012), while certain observations tend to contradict it (e.g., Byrne et al., 1992; Brown et al., 1994). However, readers are still often categorized in this way (Burt and Heffernan, 2012).

In word spelling, Weekes (1994) defined two subgroups of readers, namely lexical and non-lexical readers, and found that the lexical readers were more accurate than the non-lexical readers when spelling irregular words but that both types of readers had similar performances on non-word spelling. It is worth mentioning, however, that certain studies suggest that readers—and not subtypes of readers—might be able to (more or less) strategically control the type of processing—lexical vs. non-lexical—depending on the stimulus characteristics (e.g., Zevin and Balota, 2000)^2^. The only work we are aware of on the issue of strategic control over the lexical vs. non-lexical route in word spelling is that of Bonin et al. (2005) who found no evidence of strategic control over the non-lexical route. Finally, at the macrolevel of written *text* production, Levy and Ransdell (1995) identified individual writing profiles by analyzing transitional probabilities between the processes of planning, text generation, and reviewing and revising during different writing sessions. It is worthy of note that the research conducted on the issue of strategic control over the two routes in both word reading and spelling has been conducted at the level of groups of participants and not at the level of individuals. It is possible that individuals vary in terms of knowledge that is recruited to perform word reading and spelling tasks. This issue was recently addressed by Yap et al. (2012) in word recognition. They ran a large scale investigation of individual differences based on the lexical decision and word reading trial-levels taken from the English Lexicon Project (ELP, Balota et al., 2007). The authors found relatively high between- and within-session reliability across different sets of stimuli. Interestingly, they did not find evidence of a trade-off between sensitivity to different types of information. Instead, individuals who were more influenced by one variable (e.g., word frequency) were also more influenced by other variables (e.g., consistency). In the present study, we addressed similar issues in handwritten spelling to dictation and used certain statistical analyses that were described in Yap et al.'s (2012) study^3^.

Spelling is a less practiced skill than reading, despite the fact that the number of electronic messages sent every day has been growing steadily in recent years (Rapp and Dufor, 2011). It is therefore clearly more likely that we observe individual differences in spelling than in a more practiced skill such as word reading. At the level of text production, Levy and Ransdell (1995) found evidence for individual differences in the way participants shifted between the various writing processes. Interestingly, they found that the shifts between processes exhibited by a given writer, were stable both within a writing session and across sessions. We will explore whether such stable patterns among inviduals are also observed within and across sessions at the microlevel of word production. Contrary to Yap et al.'s (2012) findings in word recognition, it could be that spellers exhibit a greater trade-off between sensitivity to different types of information. Given that the French orthographic system is highly inconsistent (Peereman and Content, 1999)^4^, it is not unreasonable to hypothesize that certain spellers rely more on lexical knowledge (Weekes, 1994) whereas the opposite is true for other spellers. If individuals are able to control the use of the two routes, namely the lexical route, which is sensitive to word frequency, and the non-lexical route, which is sensitive to PO consistency, one prediction is that a trade-off between the word-frequency and PO consistency variables should be found. Thus, we should observe some spellers to be more sensitive to the word frequency variable and less sensitive to PO consistency (and vice versa). However, this type of trade-off between the different types of knowledge could be modulated by the level of exposure to print among participants. Yap et al. (2012) explored this issue in word recognition and found that individuals with high vocabulary knowledge had faster and more accurate word recognition performance and generally exhibited a lower level of sensitivity to the lexical characteristics of words. As far as word spelling is concerned, it is a popular belief that individuals who read a lot and possess a rich vocabulary tend to be good spellers. However, to our knowledge, there is little evidence to support such a claim. In the present study, we used a vocabulary test (Deltour, 1993) to test the hypothesis that participants with a high level of exposure to print have better spelling performances than those with a lower level of exposure. Finally, as far as the analyses on items are concerned, we expected to replicate the findings reported in our previous studies (Bonin and Méot, 2002; Bonin et al., 2004).

## Method

### Participants

A total of 80 students (66 females; mean age of 20 years) from University of Bourgogne participated in the two sessions of the experiment and were given course credits. All were native speakers of French with normal or corrected-to-normal vision and no known hearing deficit.

### Stimuli

The original stimuli consisted of 164 nouns. All the stimuli were monosyllabic words. The word stimuli were selected from the LEXOP lexical database (Peereman and Content, 1999). The statistical characteristics of the words are presented in Table 1.

**Table 1:** **Statistical characteristics of the independent variables corresponding to the items used in the multiple regression analyses**.

	**Acoustic duration (ms)**	**Orthographic length**	**Cumulative frequency**	**Frequency trajectory**	**Bigram frequency**	**Phonological neighbors**	**PO consistency**	**Imageability**	**Vocabulary test**
Min	236	2	−3.87	−2.47	210.70	0	2	2.60	7
Max	894	7	4.67	1.64	15526	30	100	4.96	33
Mean	629	4.84	0.00	0.00	4878	13.63	57.11	4.26	19.59
*SD*	128	0.93	1.90	0.63	3386	7.74	34.46	0.51	4.84

SD, standard deviation; PO, phonology-to-orthography consistency of final units (rime units). Vocabulary test scores are by participants, other measures are by items.

Objective word frequency, number of phonological neighbors and bigram frequency counts were taken from the LEXIQUE database (New et al., 2004). Child frequency measures corresponded to the cumulative frequency over grades 1–5 given by the MANULEX database (Lété et al., 2004). Cumulative frequency and frequency trajectory were computed as the sum of (or in the case of frequency trajectory, difference between) the z-scores associated with the two measures of frequency (see Bonin et al., 2004, for details). PO consistency measures were taken from the LEXOP database (Peereman and Content, 1999). We included PO consistency measures defined on rime units (VC) in the light of studies (e.g., Delattre et al., 2006) that have found strong consistency effects when this measure is used to assess adults' writing to dictation performance. Imageability norms were taken from the Bonin et al. (2003) study.

The vocabulary test taken from Deltour (1993) comprised 34 words. Each word was presented in uppercase and was followed by six other words including a synonym. For each of the 34 words, the participants had to select for the corresponding synonym.

### Apparatus

The items were recorded by a female speaker and digitized using Audacity software on a Macintosh computer. The PsyScope software (Cohen et al., 1993) was used to run the experiment on an iMacintosh. The computer controlled the presentation of the auditory items and recorded the latencies. A graphic tablet (WACOM UltraPad A5) and a contact pen (SP-401) were used to record the graphic latencies (in ms).

### Procedure

There was an interval of at least a week between the sessions, the participants were tested individually in each session. The order of the two sessions was counterbalanced across participants. In both sessions, each trial corresponded to the following events. First, a ready signal (+) was presented for 500 ms in the center of the screen. Next, the indefinite article corresponding to the forthcoming word was visually presented for 350 ms, followed by the auditory stimulus word presented through headphones. (An indefinite article was used to avoid confusion for certain words which otherwise could have been treated as verbs instead of nouns. To anticipate the results, the main effects and the interactions found in the item analyses in the current study were the same as those found in two previous studies, i.e., Bonin and Méot, 2002; Bonin et al., 2004.) The participants then had to write down the stimulus as quickly as possible on the graphic tablet using a contact pen. For each written response, a line was drawn and the participant had to position the stylus directly above the start of the line. The participants were instructed to write down a cross when they could not identify the stimulus. The time that elapsed between the onset of the spoken word and the contact of the pen with the graphic tablet was recorded by the computer. The intertrial interval was 4 s. Each experimental session started with 20 practise trials. Each session lasted about 1 h.

The vocabulary test was administered after the spelling to dictation task in session 2 and took about 5 min to complete. The participants saw a list of 34 words presented in uppercase, and then for each of these, had to choose which of the six lowercase words corresponded best to its meaning.

## Results

### Scoring of the data

Two participants, who did not return to the lab for the second session, were eliminated from the analyses. Of the 12,792 total (potentially correct) latency trials, 700 (5.5%) corresponded to errors. Among the error types, 119 (0.9%) and 209 (1.6%) were orthographic (e.g., “trian” for “train”) or phonologically plausible errors (e.g., “trein” for “train”) respectively, whereas 108 (0.8%) were other lexical responses (e.g., “wagon” for “train”). The remaining errors took the form of technical problems (123), hesitations (17), unknown or crossed out words (58 and 66). In addition, thirty-three (0.3%) latencies above 1800 ms were set apart (we did not eliminate short latencies since the shortest latency was 266 ms). Finally, latencies that were more than 2.5 standard deviations above each participant's mean were also considered as outliers [265 trials (2.1%)].

### Reliability analyses

The data for each participant were organized into sessions (Session 1 = S1 and Session 2 = S2). The trials within each session were labeled as odd and even trials depending on the alphabetical order of the items. The comparison of S1 and S2 trials made it possible to assess between-session reliability, whereas the comparison of odd and even trials permitted the assessment of within-session reliability.

The mean values of the different statistics (see Table 2) were quite similar, with similar differences being observed between sessions as well as between even-odd items. Within-session reliability scores were very high for the mean latencies and, to a lesser extent, for the standard deviations. However, the correlations were lower for the error scores. This could be due in part to the relatively restricted range of this variable. Although the same pattern was observed for between-session reliability scores, all the correlations were lower than those that were computed on the within-session scores. Yap et al. (2012) also found that between-session reliability was lower than within-session reliability in their word recognition data (however, the values of the various individual parameters that they took into account were generally higher than in our study).

**Table 2:** **(Upper) Mean percentages of errors, mean latencies and standard deviations (in ms) overall and as a function of sessions and for the odd and even trial within sessions; (Lower) Correlations between session 1 (S1) and session 2 (S2) and between odd and even trials for errors and for the means and standard deviations of the latencies**.

	**Overall**	**S1**	**S2**	**Odd**	**Even**
Errors (%)	2.57	2.29	2.83	3.34	1.78
Standard deviations of errors	2.22	2.36	2.53	2.85	2.00
Mean latencies	842.71	848.27	837.33	843.47	841.94
Standard deviations of latencies	125.59	123.93	120.34	125.32	125.68
**Correlations**		**S1-S2**		**Odd-Even**	
Errors		0.623		0.657	
*M*		0.898		0.992	
*SD*		0.635		0.859	

Errors correspond to the number of orthographic or plausible phonological errors. Means and standard deviations are those corresponding to the mean RT by subject in each subset of items.

### Correlation analyses on item means (latencies and errors)

Table 3 shows the correlations between the different variables. Phonological/orthographic spelling errors were negatively correlated with cumulative frequency, PO consistency and imageability with the result that there were fewer spelling errors on high-frequency, PO-consistent or highly-imageable words than on less frequent, consistent or less imageable words. Although the correlation between the mean error rates and the mean latencies was positive and reliable, with the result that words with longer latencies yielded more errors, it was nevertheless relatively low. Two other correlations are worth noting: (1) The mean latencies were correlated with the acoustic durations of the items, i.e., longer latencies were associated with words that had longer acoustic durations, and (2) the latencies were correlated with the cumulative frequencies of the words, with the result that the latencies were shorter for highly-frequent words than for low-frequency words.

**Table 3:** **Correlations between the variables**.

	**Latency**	**Acoustic duration (ms)**	**Orthographic length**	**Cumulative frequency**	**Frequency trajectory**	**Bigram frequency**	**PO consistency**	**Phonological neighbors**	**Imageability**
Errors	0.310^***^	−0.001	−0.050	−0.440^***^	0.134	−0.041	−0.205^***^	−0.064	−0.263^***^
Mean latency		0.523^***^	0.111	−0.339^***^	0.123	−0.083	−0.018	−0.197^**^	−0.188^*^
Acoustic duration			0.546^***^	−0.158^*^	0.141	−0.005	0.442^***^	−0.425^***^	−0.103
Orthographic length				−0.200^**^	0.110	0.296^***^	0.378^***^	−0.440^***^	−0.178^*^
Cumulative frequency					0.000	0.141	−0.085	0.236^**^	0.401^***^
Frequency trajectory						0.110	0.055	−0.076	−0.236^**^
Bigram frequency							0.082	0.127	−0.045
PO consistency								−0.342^***^	−0.073
Phonological neighbors									0.061

PO, phonology-to-orthography consistency of rime units.

*** p < 0.001;

** p < 0.01;

* *p < 0.05*.

### Regression model 1: examination of independent effects

A simultaneous regression analysis was performed with the mean latencies taken as the dependent variable and acoustic duration, number of letters, cumulative frequency, frequency trajectory, bigram frequency, phonological neighborhood, PO consistency and imageability taken as independent variables (IV)^5^. All the frequency values were log-transformed before being entered in the regression equations. Overall, the results on the latencies were consistent with those found by Bonin et al. (2004). The overall equations were reliable for latencies and errors (*R*^2^ = 0.471 and 0.298, respectively). The most important determinant of spelling latencies was acoustic duration (β = 0.733, *SE* = 0.076, *p* < 0.001). Words that had shorter acoustic durations were produced more rapidly than words with longer acoustic durations. A significant effect of word length was found (β = −0.297, *SE* = 0.081, *p* < 0.001) as were reliable effects of cumulative frequency (β = −0.283, *SE* = 0.067, *p* < 0.001) and PO consistency (β = −0.283, *SE* = 0.067, *p* < 0.001). The effect of length was negative, that is to say, for a fixed set of values of other IVs, words with more letters were produced faster than those with less letters. Although at first glance, this result is somewhat counter-intuitive, it is the same as that reported in the Bonin et al. (2004) study. Also, words with low word frequency/PO consistency values took longer to write down than words with higher word frequency/PO consistency values.

As far as errors are concerned, the multiple regression analysis revealed significant effects of cumulative frequency (β = −0.440, *SE* = 0.077, *p* < 0.001) and PO consistency (β = −0.262, *SE* = 0.077, *p* < 0.001) (Table 4). There were more errors on words of low frequency/PO consistency than on words of high frequency/PO consistency. We performed two separate analyses on (1) phonologically plausible errors and (2) purely orthographic errors. The results of these two analyses were similar except that, in addition to the significant effects of cumulative frequency and PO consistency, there were also effects of word length and frequency trajectory on the number of phonologically plausible errors.

**Table 4:** **Correlations of the by-subject independent variables' effects**.

		**2**	**3**	**4**	**5**	**6**	**7**	**8**
1	Acoustic duration	−0.33^**^	−0.21^†^	0.16	0.04	−0.46^***^	0.06	0.02
2	Orthographic length		0.43^***^	−0.10	−0.46^***^	−0.16	0.25^*^	0.02
3	Cumulative frequency			−0.21^†^	−0.24^*^	−0.07	0.11	−0.09
4	Frequency trajectory				−0.12	−0.18	0.00	0.32^**^
5	Bigram frequency					0.05	−0.22^*^	−0.15
6	PO consistency						0.03	0.07
7	Phonological neighbors							0.11
8	Imageability							1

*** p < 0.001;

** p < 0.01;

* p < 0.05;

† < 0.1.

### Regression model 2: examination of the interaction term of PO consistency, cumulative frequency and imageability

When the interaction term of cumulative frequency, PO consistency and imageability was included in the multiple regression model, it was significant both on latencies and error rates (β = −0.138, *SE* = 0.066, *p* < 0.05 and β = −0.252, *SE* = 0.067, *p* < 0.001). On errors only, the interaction terms of cumulative frequency and PO consistency (β = 0.276, *SE* = 0.071, *p* < 0.001) and of cumulative frequency and imageability (β = 0.249, *SE* = 0.066, *p* < 0.001) were significant. On both latencies and error rates, the independent effects of orthographic length (β = −0.306, *SE* = 0.081, *p* < 0.001 and β = −0.18, *SE* = 0.082, *p* < 0.03 respectively), cumulative frequency (β = −0.309, *SE* = 0.07, *p* < 0.001; β = −0.466, *SE* = 0.072, *p* < 0.001) and PO consistency (β = −0.24, *SE* = 0.07, *p* < 0.001; β = −0.161, *SE* = 0.071, *p* < 0.05) were reliable. On latencies only, there was a significant effet of acoustic duration (β = 0.725, *SE* = 0.077, *p* < 0.001).

The facilitatory effect of PO consistency was larger for items having low frequency and low imageability values. Indeed, given that (1) The tests of the simple effects of PO consistency at these levels (i.e., low frequency and low imageability levels) were reliable for both latencies and errors, *t*_(151)_ = −4.62 *p* < 0.001 and *t*_(151)_ = −7.18. *p* < 0.001, respectively and (2) The simple PO consistency effect also reached significance only for the latencies on high frequency and high imageability words, [*t*_(151)_ = −2.23, *p* < 0.05], it can be seen that the influence of PO consistency is specific to words of low imageability and of low word frequency.

### Distributions of standardized regression coefficients across participants

**Figure 1:**
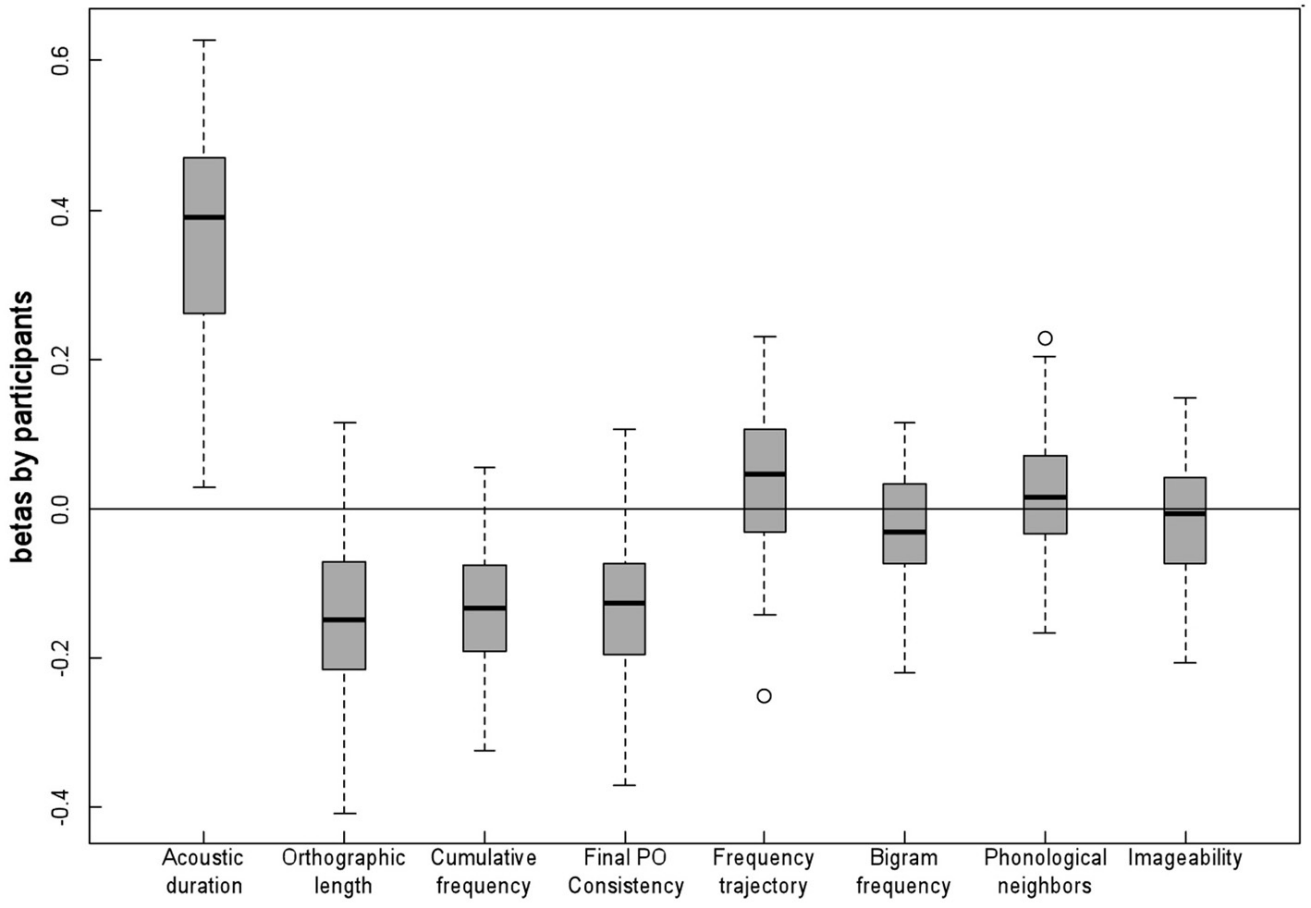
**Distributions of the by-participants' standardized regression coefficients**.

For each participant, we conducted simultaneous regression analyses with the latencies taken as the dependent variable and the same independent variables as used in Regression 1. Figure 1 presents the distributions of the resulting standardized regression coefficients. There were four aspects of note. First of all, there was a substantial variability in the magnitude of the effects that were produced by the participants. For example, although virtually all participants produced negative regression coefficients for the word frequency effect (see Figure 1), with faster latencies on higher frequency words than on lower frequency words, the coefficients varied between −0.32 and +0.06. Second, the direction and relative magnitudes of participants' level effects were generally consistent with the items' level effects reported above. In effect, acoustic duration was the best predictor, followed by word frequency, PO consistency and number of letters. Third, the distributions of the coefficients were roughly symmetric, with no noticeable skew. Fourth, the variability associated with the acoustic duration and the number of letters variables was clearly higher than for the other independent variables.

### Between and within-sessions reliability of the individual effects of the independent variables

The inter-session reliabilities of the betas and R-squares by participants were low (correlations varied between −0.08 and 0.14). Although somewhat higher, the within-session reliabilities were also not high (correlations varied between −0.06 and 0.57). The correlations between odd and even items were among the highest for three of the independent variables which were found to be the most important predictors in the by-items analysis, namely acoustic duration (0.48, *p* < 0.001), orthographic length (0.33, *p* < 0.01) and PO consistency (0.23, *p* < 0.05). This was not the case for cumulative frequency for which the correlation was nearly zero. Moreover, the bigram frequency effects were also significantly correlated within sessions (0.29, *p* < 0.01).

### Correlations between the by-participant's effects of the independent variables

As can be seen from Table 4, the correlations between the by-participants standardized regression coefficients for pairs of independent variables revealed that the higher the effect of acoustic duration was, the lower the effects of PO final consistency and cumulative frequency were. The participants who were the most sensitive to the acoustic duration variable were also those who benefited the most from higher words PO consistency and cumulative frequency values. The same relationship was found with orthographic length. There were also positive correlations between the coefficients of orthographic length and cumulative word frequency (that is to say between two of the variables having facilitatory effects) and the finding that the participants who were the most sensitive to one of these variables also tended to be more affected by the other. Importantly, the correlation between cumulative word frequency and PO consistency was low and not reliable. Two other significant correlations are worth mentioning, namely those between bigram frequency and orthographic length, and between frequency trajectory and imageability.

### Relations between vocabulary knowledge and effects of different psycholinguistic variables

As shown in Table 5, mean error rates (including both phonologically plausible errors and orthographic errors) and mean latencies were reliably and negatively correlated with vocabulary knowledge. High vocabulary scores were associated with shorter latencies, fewer errors. Surprisingly, the correlation between the number of errors and the mean latencies was not reliable.

**Table 5:** **Correlations between the by subject independent variables' effects (rows) and vocabulary test scores, mean latencies, numbers of errors and correct spellings**.

	**Vocabulary test**	**Latency**	**Errors**
Errors	−0.424^***^	0.196	
Latency	−0.309^**^		
R-square	0.172	−0.263^*^	−0.058
Acoustic duration	0.229^*^	−0.380^***^	−0.155
Orthographic length	−0.229^*^	0.132	0.089
Cumulative frequency	0.020	0.008	−0.051
Frequency trajectory	0.073	−0.073	−0.130
Bigram frequency	0.018	0.151	0.072
PO consistency	0.017	0.158	−0.009
Phonological neighbors	0.045	0.057	0.033
Imageability	0.054	−0.216	−0.101

*** p < 0.001;

** p < 0.01;

* p < 0.05.

**Figure 2:**
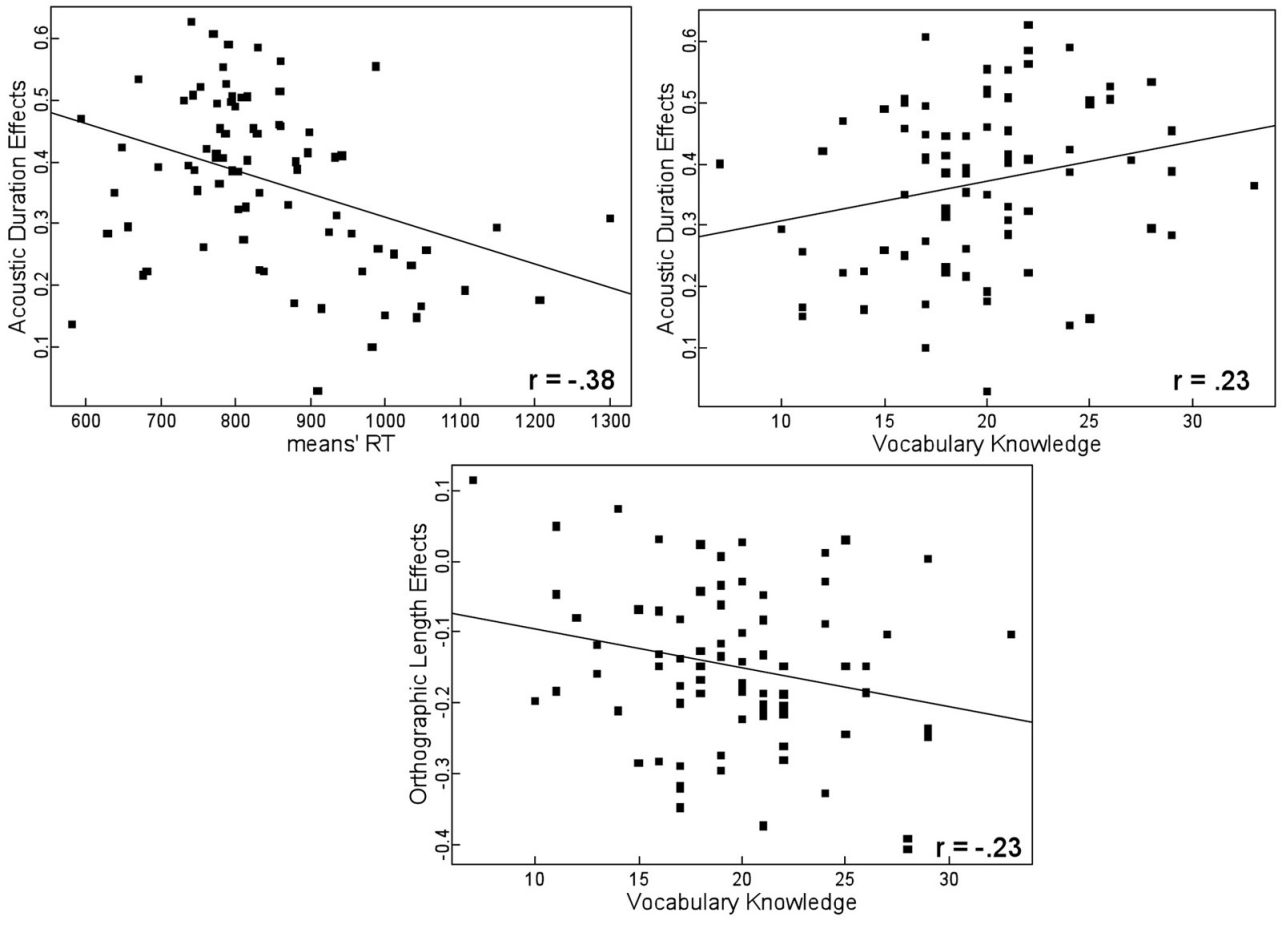
**Relations between participants' performance, vocabulary knowledge and acoustic duration, and orthographic length effects**.

Turning to the relationships between the effects of the psycholinguistic variables and the characteristics of the participants, the first aspect worth mentioning is that the amount of explained variance was negatively correlated with the mean latencies by participants. In other words, the fastest participants were those whose psycholinguistic variables best explained their latencies. Second, although the correlation between R-squares and vocabulary knowledge scores was positive (a finding that is consistent with the negative correlation found between mean latencies and vocabulary knowledge), it nevertheless failed to reach significance. Not surprisingly, the correlation between the mean latencies and the effects of acoustic duration was negative, suggesting that the more sensitive the participants were to the acoustic duration variable, the smaller their mean latencies were. Accoustic duration effects were also positively correlated with vocabulary knowledge scores, that is to say, the participants with greater vocabulary knowledge also exhibited higher acoustic duration effects (Figure 2). By contrast, orthographic length effects and vocabulary knowledge were negatively correlated: the participants with greater/lower vocabulary knowledge exhibited lower/greater length effects (Figure 2).

## Discussion

In the present study, we examined several issues relating to individual differences in handwritten spelling to dictation in addition to providing important findings at the level of items. Adult participants had to write down 164 auditorily presented words in two different sessions. Handwritten spelling onset latencies and error rates on words were recorded. We observed a number of important and novel findings that have implications for models of the spelling process.

First of all, the analyses on the item means led us to identify several reliable predictors of spelling speed and error rates (see below). These were generally consistent with previously reported findings (Bonin and Méot, 2002; Bonin et al., 2004). Second, we found that both within and between-sessions reliabilities were relatively high for the mean latencies and their standard deviations, but less so for error scores. Third, it was not possible to identify different types of spellers on the basis of their reliance on one of the two routes: lexical vs. non-lexical. Fourth, we found that the level of vocabulary knowledge in individuals affected both overall spelling performance as well as specific aspects of it. These findings are now discussed in turn.

Not surprisingly, but importantly, the multiple regression analyses performed on mean spelling latencies for items replicated previous findings (e.g., Bonin and Méot, 2002; Bonin et al., 2004). The main determinants of handwritten latencies were acoustic duration, orthographic length, cumulative word frequency and PO consistency. As far as error rate is concerned, cumulative frequency and PO consistency had a reliable influence. In addition the interaction between cumulative word frequency, PO consistency and imageability was reliable in both spelling latencies and error rates (two two-way interactions were also reliable on this latter measure). The finding that word frequency and PO consistency exert an effect on both latencies and errors accords with the dual-route view of spelling to dictation, according to which both types of knowledge contribute to the building of orthographic codes. These findings strongly suggest that the non-lexical route is not an optional route but is instead involved in the spelling of familar words (Kreiner and Gough, 1990).

Words having long acoustic durations were initialized later than words having shorter durations. This finding suggests that participants start writing down the words when they have fully understood them. Since the processing of the auditory string is necessarily distributed over time, words which take more time to be fully heard take longer to process than those that take less time to be fully heard, and this delay is reflected in the spelling latencies. Furthermore, orthographic length also had a non-trivial influence on spelling latencies. As found by Bonin and Méot (2002), the longer the words, the shorter the latencies. Indeed, in visual word recognition, the influence of the number of letters has also been found to have a non-trivial (and somewhat complex) relationship with lexical decision times (see Ferrand et al., 2010).

Turning to the frequency trajectory and imageability variables, we did not find that these variables made a reliable independent contribution to spelling latencies. In the Bonin et al. (2004) study, no influence of frequency trajectory on spelling to dictation latencies was predicted since these effects are generally found in tasks which involve arbitrary mappings such as object or face naming and not in tasks such as spelling to dictation or word naming which involve quasi-regular mappings in both French and English (see Mermillod et al., 2012 for a full discussion). Although imageability was not found to make an independent contribution, it interacted reliably with word frequency and PO consistency. This interaction was also reported by Bonin and Méot (2002) and it is in line with the dual-route view of spelling to dictation. The Cumulative word frequency × PO consistency × Imageability interaction shows that the joint influence of word frequency and PO consistency is most specifically observed on words of low imageability. Although this type of interaction has been reported in word reading aloud (Strain et al., 1995), its reliability has been disputed (see Monaghan and Ellis, 2002). The interaction between word frequency and PO consistency in spelling to dictation latencies is not a novel finding since it was reported by Bonin and Méot (2002). This outcome is consistent with the prediction of the dual-route view of spelling to dictation. In this framework, consistency effects arise due to a competition between the outcomes of the lexical and non-lexical routes, respectively (Rapp et al., 2002). In the case of inconsistent words, the lexical route produces a correct orthographic code based on lexical activation that competes against an incorrect code assembled by the non-lexical route. More precisely, for inconsistent words, the individual graphemes activated by the lexical pathway compete for selection with those assembled from the non-lexical pathway and the resolution of this conflict takes some time. According to Rapp et al. (2002), the cognitive spelling system has an interactive architecture incorporating feedback between individual graphemes and orthographic wordform representations. This bidirectional connectivity between orthographic wordform representations and individual graphemes in spelling to dictation permits the activation coming from the lexical route to prevail over the activation from the non-lexical route (Rapp et al., 2002). The finding that a PO consistency effect was observed primarily for low frequency/low imageability words suggests that the strength of the orthographic lexical code is determined not only by the frequency of the item, but also by its semantic richness, since imageability is thought to index semantic code activation (Evans et al., 2012).

Turning to the analyses performed at the participants level, we found the participants' mean latencies (and standard deviations) were highly reliable both within and between-sessions. We think that this finding is a good news for researchers who want to study spelling performance based on the use of groups of participants generally tested within a single session. In the Yap et al. (2012) study, the reliability of both lexical decision and word naming times were also found to be high. The reliability scores were, however, lower for the errors (both within and between sessions). This might, in part, have been due to the relatively restricted ranges of this variable. This latter finding could also be attributable to the fact that participants' orthographic knowledge is fragile. Since the French orthographic system is highly inconsistent, it is difficult to master the spellings of many words. Most of them have to be learned and stored in long-term memory in order to be produced correctly. This could therefore explain why the error scores on one set of words do not correlate strongly with error scores on other sets of words.

As reviewed in the Introduction, the idea that there are different types of spellers originates from certain studies in the literature on word reading (e.g., Baron and Strawson, 1976), on the one hand, and on written text production (Levy and Ransdell, 1995), on the other. It has been suggested that there are different types of readers who differ at the level of the pathway they predominantly use. For instance, the early work of Baron and Strawson (1976) suggested that *Phoenician* readers predominantly use the non-lexical route while *Chinese* readers predominantly use the lexical route. Although the hypothesis of different types of readers is appealing, it lacks solid empirical support and a recent investigation in adults failed to provide evidence to back up the hypothesis that there are different groups of readers who rely more extensively on either the lexical or the non-lexical route during word spelling (Burt and Heffernan, 2012). In our study, we also tested whether there were individual differences among spellers in their mobilization of the two processing pathways (lexical vs. non-lexical) in word spelling to dictation. The dual-route view of word spelling holds that the lexical route directly maps semantic codes corresponding to spoken words onto underlying lexical orthographic representations and the non-lexical route assembles the spelling of words on the basis of spelling-to-sound mappings (Rapp et al., 2002). We hypothesized that if individuals are able to control the extent to which they use the two routes, one sensitive to frequency (the lexical route) and the other sensitive to PO consistency (the non-lexical route), a trade-off between the word-frequency and PO consistency variables should be observed. In other words, we expected that spellers who were more sensitive to the word frequency variable would be less sensitive to PO consistency (and vice versa).

The analyses performed on the correlations between the by-participant effects of the independent variables did not reveal any trade-off between the lexical and non-lexical routes. In particular, there was no reliable correlation between the PO consistency and cumulative frequency variables. It should be remembered that Yap et al. (2012) did not find this type of trade-off in their lexical decision and word naming tasks. The current data therefore provides evidence against the idea that spellers differ at the level of their use of lexical vs. non-lexical information. However, we hypothesized that this type of relationship could be modulated by the level of exposure to print. (The relationships between level of exposure to print and sensitivity to the different variables will be discussed in more detail below.) Moreover, the analyses revealed that there was a positive correlation between the coefficients of two variables which exert facilitatory effects on spelling performance (word frequency and orthographic length), with the result that the participants who were the most sensitive to one of these variable also tended to be more strongly affected by the other.

Finally, the examination of the distributions of standardized regression coefficients revealed that there was a substantial variability in the magnitude of the effects that were produced by the participants. Take, for instance, the case of word frequency which has been reported in various spelling to dictation studies involving participant goup-level (e.g., Delattre et al., 2006) or item-level (e.g., Bonin et al., 2004) analyses. We found that, even though the regression coefficients for the effect of word frequency were negative for the majority of the participants, these coefficients were nevetherless subject to a certain level of variability. These individual analyses contribute to our understanding of how the effects of the different variables do or do not correspond to those obtained at the level of groups of participants or at the level of items. In the present study, the direction and relative magnitudes of the effects observed at the level of individual participants were generally consistent with those observed in the by-items analyses. One surprising aspect of the analyses on individual differences was that the inter-session reliabilities of the betas and R-squares corresponding to the different independent variables were low. This suggests that the participants were quite flexible in the way they mobilized the different processes and representations involved in spelling words to dictation. This flexibility cannot be evaluated when the analyses are restricted to the means measured at the level of groups of participants or items.

We used the vocabulary test designed by Deltour (1993), to index the level of exposure to print. In word recognition, Yap et al. (2012) have put forward the hypothesis that vocabulary knowledge could reflect the integrity of underlying lexical representations. Their idea is that participants with a high level of vocabulary knowledge possess lexical representations that are of better quality than participants with poor vocabulary knowledge. Indeed, it is generally believed that good spellers possess lexical orthographic representations that are of better quality (that is to say, that are more accurate, easier to retrieve and more interconnected). Good spellers should therefore have more integrated orthographic representations than poor spellers due to their more extensive exposure to reading material. Consequently, they should have stored more words with accurate orthographies in long-term memory. The idea that reading a lot enhances orthographic knowledge that is used in spelling is further supported by several studies suggesting that the orthographic representations used in reading and spelling are shared (e.g., Rapp and Lipka, 2011). We therefore hypothesized that participants with a high level of exposure to print would possess orthographic representations of better quality than those with a low level of exposure to print.

We indeed found that individuals who achieved high vocabulary scores wrote down the words more quickly and made fewer spelling errors (including both phonologically plausible errors and orthographic errors), and were therefore more accurate, than those who had poorer vocabulary scores. A somewhat surprising finding was that the number of errors and the mean latencies were not reliably correlated. We found that the slower the individuals were, the lower their vocabulary score was, and conversely, that the faster they were, the higher their score in the vocabularity test was. The findings obtained for spelling are consistent with the Yap et al. (2012) study which also found that word recognition performance was better in participants having high vocabulary knowledge Furthermore, the faster (slower) they were, the greater (or lesser) the extent to which their RTs were accounted for by the independent variables.

Turning to the effect of psycholinguistic variables and vocabulary knowledge, the prediction with respect to vocabulary size was that there would be a greater reliance on the lexical route in spelling. The correlation between the R-squares of the psycholinguistic variables and the scores in the vocabulary test was positive but failed to reach significance. In word recognition, Yap et al. (2012) found that greater vocabulary knowledge was associated with a generally reduced level of sensitivity to underlying lexical characteristics. According to them, this was due to the fact that the lexical decision task requires participants to discriminate between words and non-words and that participants with good vocabulary knowledge have different activation thresholds for low-frequency words than those with poor vocabulary knowledge.

We found that the participants who had the highest scores in the vocabulary test exhibited stronger effects of acoustic duration than those who had the lowest scores in this test. (The same relationship was found with the participants' mean spelling speed, with the result that the slowest participants exhibited the weakest effect of acoustic duration whereas the fastest participants exhibited the strongest effect of this variable.) We consider that the influence of acoustic duration on spelling latencies is attributable to the word identification-comprehension stage. Since the auditory signal corresponding to words is spread out over time, words that take a long time to process take also more time to be understood than shorter words.

In contrast, orthographic word length had a negative influence on spelling latencies which were shorter with longer words. The fact that the participants who scored higher in the vocabulary test also exhibited a stronger effect of word length suggests that participants with a higher vocabulary level initiate the spelling of longer words faster than those with a lesser degree of vocabulary knowledge. As far as the effects of orthographic word length are concerned, Rapp and Dufor (2011) have argued that these effects in the spelling performance of patients are due to impairments at the level of the orthographic working memory system. This memory system keeps individual graphemes active before they are selected for further processing. The finding that adults with good vocabulary knowledge exhibited a stronger positive effect of the acoustic duration variable, but at the same time, a stronger negative effect of the number of letters, suggests that they process the input more thoroughly before initiating the first handwriting movement than adults with less vocabulary knowledge. This suggests that when spelling, the former may take longer time to identify and understand the words, and maybe also to perform an internal verification of their spellings, before starting to write. This strategy can be beneficial since, for the same level of acoustic duration, high vocabulary knowledge participants take less time to start writing longer words than shorter words. It is possible that because spellers with less vocabulary knowledge possess less accurate orthographic representations (and are certainly aware that they lack orthographic knowledge), their processing is not only shallower (they may perform less internal monitoring) but also more serial in nature in order to permit them to monitor their spelling while writing the different letters on the sheet of paper. The specific relationships between the variables of acoustic duration and orthographic word length, on the one hand, and vocabulary knowledge scores, on the other, are somewhat complex and we must acknowledge that we did not predict any such relationship. Further research will be required to determine just how it is possible to account for these relationships.

To conclude, we are aware that one limitation of our study is that we focused on monosyllabic words and that future work should investigate words of all types. However, we hope to have shown convincingly that, in addition to the more traditional approach at the level of items, the use of the multiple regression approach to investigate individual differences in adult word spelling can contribute to a better understanding of the mechanisms and representations that are involved in this complex human skill.

### Conflict of interest statement

The authors declare that the research was conducted in the absence of any commercial or financial relationships that could be construed as a potential conflict of interest.

## Footnotes

^1^PO consistency is a measure of the ambiguity of sound and spelling mappings. For instance, phoneme-grapheme consistency takes into account both the frequency with which a particular phoneme is associated with a particular grapheme and the overall frequency of the grapheme whatever its pronunciation. When the phoneme is always associated with the same grapheme, the ratio is equal to 1. When multiple associations exist, the ratio is less than 1. PO consistency values vary between 0 and 1.

^2^However, this issue has been the cause of some debate since the evidence put forward to support a strategic modulation of the processing pathways has also been taken to support an alternative account (i.e., the time-criterion account, see for instance Chateau and Lupker, 2003).

^3^The Yap et al. (2012) statistical approach can be seen as an extension of that of Lorch and Myers (1990). The difference is that the tests are not only performed in order to compare the means of the participants' effects to zero but also to compute correlational tests between these effects and certain characteristics of the participants (e.g., vocabulary scores).

^4^Like English, the French language is characterized by the presence of ambiguous phoneme-grapheme mappings. Estimates indicate greater consistency in French than in English in the orthography-to-phonology direction but reduced consistencies in the phonology-to-orthography direction in both languages (Peereman and Content, 1998). In particular, in spelling many inconsistencies are located at word endings in French. For example the /aR/ unit can be spelled in at least three different ways, the most frequent rendering being “are” (28.12) and the two next most frequent being “ar” and “ard,” both of which have similar consistency scores (18.75). The difference in consistency scores is not high between the different orthographic renderings of the same phonological unit and this renders the categorization of the words in terms of “regular” and “irregular” difficult.

^5^As the IVs' greatest VIF was below 2 for all IVs (even when including interaction terms), we consider that multicolinearity problems could not have drastically affected the results of the regression analysis including all variables simultaneously.

## References

[B2] Balota D. A. Yap M. J. Cortese M. J. Hutchison K. A. Kessler B. Loftis B. (2007). The English lexicon project. Behav. Res. Methods 39, 445–459 10.3758/BF03193014 17958156

[B3] Baron J. Strawson C. (1976). Use of orthographic and word-specific knowledge in reading words aloud. J. Exp. Psychol. Hum. Percept. Perform. 2, 386–393 10.1037/0096-1523.2.3.386

[B4] Bonin P. Barry C. Méot A. Chalard M. (2004). The influence of age of acquisition in word reading and other tasks: a never ending story. J. Mem. Lang. 50, 456–476 10.1016/j.jml.2004.02.001

[B5] Bonin P. Collay S. Fayol M. Méot A. (2005). Attentional strategic control over sublexical and lexical processing in written spelling to dictation in adults. Mem. Cogn. 33, 59–75 10.3758/BF03195297 15915793

[B6] Bonin P. Méot A. (2002). Writing to dictation in real time in adults: what are the determinants of written latencies?, in Advances in Psychology Research, Vol. 16, ed Shohov S. P. (New York, NY: NovaScience Publishers), 139–165

[B7] Bonin P. Méot A. Aubert L. Malardier N. Niedenthal P. Capelle-Toczek M. C. (2003). Normes de concrétude, de valeur d'imagerie, de fréquence subjective et de valence émotionnelle pour 867 mots [Concreteness, imageability, subjective frequency and emotional valence norms for 867 words]. L'Année Psychol. 104, 655–694 10.3406/psy.2003.29658

[B8] Brown P. Lupker S. J. Colombo L. (1994). Interacting sources of information in word naming: a study of individual differences. J. Exp. Psychol. Hum. Percept. Perform. 20, 537–554 10.1037/0096-1523.20.3.537

[B9] Burt J. E. Heffernan M. E. (2012). Reading and spelling in adults: are there lexical and sub-lexical subtypes. J. Res. Read. 35, 183–203 10.1111/j.1467-9817.2010.01455.x

[B10] Byrne B. Freebody P. Gates A. (1992). Longitudinal data on the relations of word-reading strategies to comprehension, reading time, and phonemic awareness. Read. Res. Q. 27, 140–151 10.2307/747683

[B11] Chateau D. Lupker S. J. (2003). Strategic effects in word naming: examining the route emphasis versus time-criterion accounts. J. Exp. Psychol. Hum. Percept. Perform. 29, 139–151 10.1037/0096-1523.29.1.139 12669753

[B12] Cohen J. McWhinney B. Flatt M. Provost J. (1993). PsyScope: an interactive graphic system for designing and controlling experiments in the psychology laboratory using Macintosh computers. Behav. Res. Methods Instrum. Comput. 25, 257–271 10.3758/BF03204507

[B13] Coltheart M. Rastle K. Perry C. Langdon R. Ziegler J. (2001). DRC: a dual route cascaded model of visual word recognition and reading aloud. Psychol. Rev. 108, 204–256 10.1037/0033-295X.108.1.204 11212628

[B15] Delattre M. Bonin P. Barry C. (2006). Written spelling to dictation: do irregularity effects persist on writing durations. J. Exp. Psychol. Learn. Mem. Cogn. 32, 1330–1340 10.1037/0278-7393.32.6.1330 17087587

[B16] Deltour J. J. (1993). Echelle de vocabulaire de Mill Hill, in Adaptation Française et Normes Europeennes du Mill Hill et du Standard Progressive Matrices de Raven (PM38), ed Raven de J. C. (Braine-le-Chateau: Editions l'application des techniques modernes).

[B17] Evans G. Lambon Ralph M. Woollams A. (2012). What's in a word. A parametric study of semantic influences on visual word recognition. Psychon. Bull. Rev. 19, 325–331 10.3758/s13423-011-0213-7 22258820

[B18] Ferrand L. New B. Brysbaert M. Keuleers E. Bonin P. Méot A. (2010). The french lexicon project: lexical decision data for 38, 840 French words and 38, 840 pseudowords. Behav. Res. Methods 42, 488–496 10.3758/BRM.42.2.488 20479180

[B19] Kreiner D. S. Gough P. B. (1990). Two ideas about spelling: rules and word-specific memory. J. Mem. Lang. 29, 103–118 10.1016/0749-596X(90)90012-O

[B20] Lété B. Sprenger-Charolles L. Colé P. (2004). MANULEX: a grade-level lexical database from French elementary-school readers. Behav. Res. Methods Instrum. Comput. 36, 156–166 10.3758/BF03195560 15190710

[B21] Levy M. C. Ransdell S. (1995). Is writing as difficult as it seems. Mem. Cogn. 23, 767–779 10.3758/BF03200928 8538448

[B22] Lorch R. F. Myers J. L. (1990). Regression analyses of repeated measures data in cognitive research. J. Exp. Psychol. Learn. Mem. Cogn 16, 149–157 10.1037/0278-7393.16.1.149 2136750

[B25] Mermillod M. Bonin P. Méot A. Ferrand L. Paindavoine M. (2012). Computational evidence that frequency trajectory theory does not oppose but emerges from age-of-acquisition theory. Cogn. Sci. 36, 1499–1531 10.1111/j.1551-6709.2012.01266.x 22985438

[B26] Monaghan J. Ellis A. W. (2002). What exactly interacts with spelling-sound consistency in word naming. J. Exp. Psychol. Learn. Mem. Cogn. 28, 183–206 10.1037/0278-7393.28.1.183 11827080

[B28] New B. Pallier C. Brysbaert M. Ferrand L. (2004). Lexique 2: a new french lexical database. Behav. Res. Methods Instrum. Comput. 36, 516–524 10.3758/BF03195598 15641440

[B29] Paap K. R. Noel R. W. (1991). Dual-route models of print to sound: still a good horse race. Psychol. Res. 53, 13–24 10.1007/BF00867328

[B30] Peereman R. Content A. (1998). Quantitative Analyses of Orthography to Phonology Mapping in English and in French. Available online at: http://homepages.ulb.ac.be/~acontent/OPMapping.html

[B31] Peereman R. Content A. (1999). LEXOP: a lexical database providing orthographyphonology statistics for French monosyllabic words. Behav. Res. Methods Instrum. Comput. 31, 376–379 10.3758/BF03207735 10495825

[B32] Purcell J. Turkeltaub P. Eden G. Rapp B. (2011). Examining the central and peripheral processes of written word production through meta-analysis. Front. Psychol. 2:239 10.3389/fpsyg.2011.00239 22013427 PMC3190188

[B33] Rapp B. Dufor O. (2011). The neurotopography of written word production: an fMRI investigation of the distribution of sensitivity to length and frequency. J. Cogn. Neurosci. 23, 4067–4081 10.1162/jocn_a_00109 21812571

[B34] Rapp B. Epstein C. Tainturier M. J. (2002). The integration of information across lexical and sublexical processes in spelling. Cogn. Neuropsychol. 19, 1–29 10.1080/0264329014300060 20957529

[B35] Rapp B. Lipka K. (2011). The literate brain: the relationship between reading and spelling. J. Cogn. Neurosci. 23, 1180–1197 10.1162/jocn.2010.21507 20433242 PMC3106999

[B36] Roux J. S. McKeeff T. J. Grosjacques G. Afonso O. Kandel S. (2013). The interaction between central and peripheral processes in handwriting production. Cognition 127, 235–241 10.1016/j.cognition.2012.12.009 23454797

[B37] Strain E. Patterson K. E. Seidenberg M. S. (1995). Semantic effects in single-word naming. J. Exp. Psychol. Learn. Mem. Cogn. 21, 1140–1154 10.1037/0278-7393.21.5.1140 8744959

[B40] Tainturier M. J. Rapp B. (2001). The spelling process, in The Handbook of Cognitive Neuropsychology: What Deficits Reveal About the Human Mind, ed Rapp B. (Philadelphia, PA: Psychology Press), 263–289

[B41] Weekes B. S. (1994). Spelling skills of lexical readers. Br. J. Psychol. 85, 245–257 10.1111/j.2044-8295.1994.tb02521.x

[B42] Yap M. J. Balota D. A. Sibley D. E. Ratcliff R. (2012). Individual differences in visual word recognition: insights from the english lexicon project. J. Exp. Psychol. Hum. Percept. Perform. 38, 53–79 10.1037/a0024177 21728459 PMC3193910

[B43] Zevin J. D. Balota D. A. (2000). Priming and attentional control of lexical and sublexical pathways during naming. J. Exp. Psychol. Learn. Mem. Cogn. 26, 121–135 10.1037/0278-7393.26.1.121 10682293

